# Button Battery Foreign Bodies in Children: Hazards, Management, and Recommendations

**DOI:** 10.1155/2013/846091

**Published:** 2013-07-11

**Authors:** Mohammed Hossam Thabet, Waleed Mohamed Basha, Sherif Askar

**Affiliations:** ^1^Department of Oto-Rhino-Laryngology and Head and Neck Surgery, Faculty of Medicine, Alexandria University, Alexandria 21526, Egypt; ^2^Department of Oto-Rhino-Laryngology and Head and Neck Surgery, Faculty of Medicine, Zagazig University, Zagazig 44519, Egypt

## Abstract

*Objective*. The demand and usage of button batteries have risen. They are frequently inadvertently placed by children in their ears or noses and occasionally are swallowed and lodged along the upper aerodigestive tract. The purpose of this work is to study the different presentations of button battery foreign bodies and present our experience in the diagnosis and management of this hazardous problem in children. *Patients and Methods*. This study included 13 patients. The diagnostic protocol was comprised of a thorough history, head and neck physical examination, and appropriate radiographic evaluation. The button batteries were emergently extracted under general anesthesia. *Results*. The average follow-up period was 4.3 months. Five patients had a nasal button battery. Four patients had an esophageal button battery. Three patients had a button battery in the stomach. One patient had a button battery impacted in the left external ear canal. Apart from a nasal septal perforation and a tympanic membrane perforation, no major complications were detected. *Conclusion*. Early detection is the key in the management of button battery foreign bodies. They have a distinctive appearance on radiography, and its prompt removal is mandatory, especially for batteries lodged in the esophagus. Physicians must recognize the hazardous potential and serious implications of such an accident. There is a need for more public education about this serious problem.

## 1. Introduction

The use of small button batteries can be attributed to the advent as well as the reduction in size of many technological devices. Button batteries are increasingly used in devices such as hearing aids, electronic games, watches, digital planners, and new electronic gadgets. Their smooth and shiny appearance makes them quite attractive and interesting to children who eagerly handle them when they are accessible [1]. Button battery foreign bodies may have a fatal outcome [2, 3]. Conversely, they may result in little to no ill effect on the child [4]. 

The clinical course of a child with a button battery depends on several factors, including the location, duration of mucosal or skin exposure, remaining voltage in the battery, and chemical composition of the battery [1]. 

The purpose of this work is to study the different presentations of button battery foreign bodies and present our experience in the diagnosis and management of this hazardous problem in children.

## 2. Patients and Methods

This study included 13 patients with a history or suspicion of a foreign body. All the patients were managed through the Department of Oto-Rhino-Laryngology and Head and Neck Surgery of Zagazig University Hospitals during the period from March 2008 to March 2011. Four patients were girls, and nine patients were boys. Their age ranged from 23 to 53 months with an average age of 36.1 months (Table 1). The Institutional Reviewer Board (IRB) of the Faculty of Medicine, Zagazig University, Zagazig, Egypt, approved the study. All the patients underwent the following diagnostic protocol: (1) a thorough history was taken for each patient with attention for the duration of foreign body impaction and symptoms such as nasal discharge, epistaxis, nasal blockage, otorrhea, otalgia, ear fullness, breathing difficulty, drooling, dysphagia, vomiting, chocking, and cough; (2) proper head and neck physical examination, including otomicroscopy and flexible nasopharyngoscopy; (3) appropriate radiographic evaluation: plain X-ray of the neck, chest, and abdomen for a swallowed foreign body and of the nose for a nasal foreign body. A fully informed written consent was obtained from the parents of all the patients. The button batteries were emergently extracted under general anesthesia. We used rigid pharyngoesophagoscopy for esophageal button batteries and nasal endoscopy for nasal button batteries.

## 3. Results

This study was conducted on 13 patients with accidental button battery foreign body ingestion or impaction in different locations. They presented two hours to one week after introduction of the foreign body. Five patients had a nasal button battery. Four patients had an esophageal button battery. Three patients had a button battery in the stomach. One patient had a button battery impacted in the left external ear canal (Table 1). 

**Figure 1:**
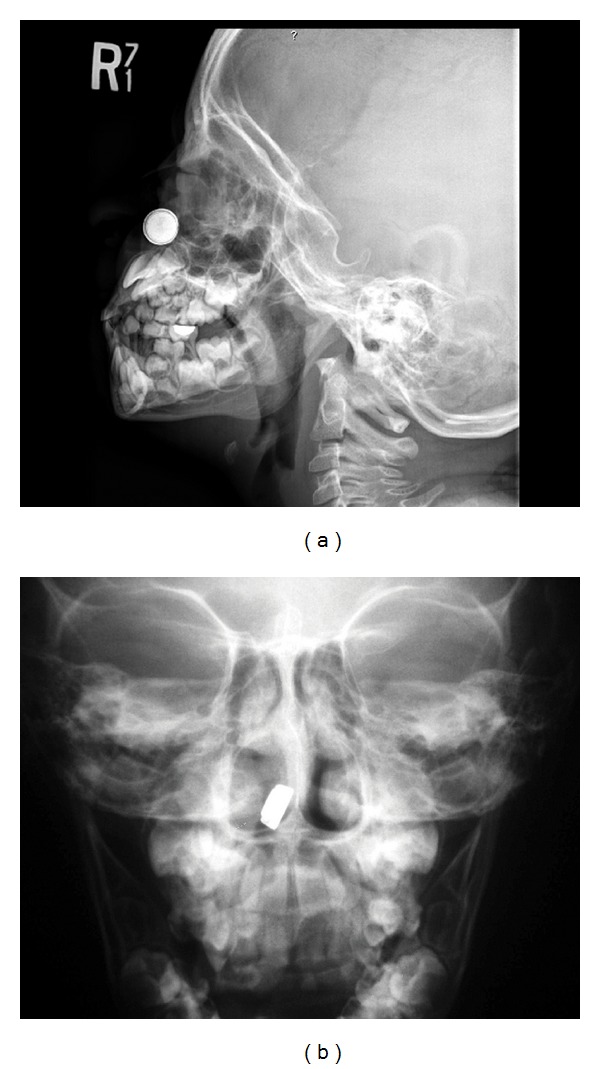
Plain X-ray: a right nasal button battery foreign body. (a) Lateral view (double contour), (b) Anteroposterior view.

**Figure 2:**
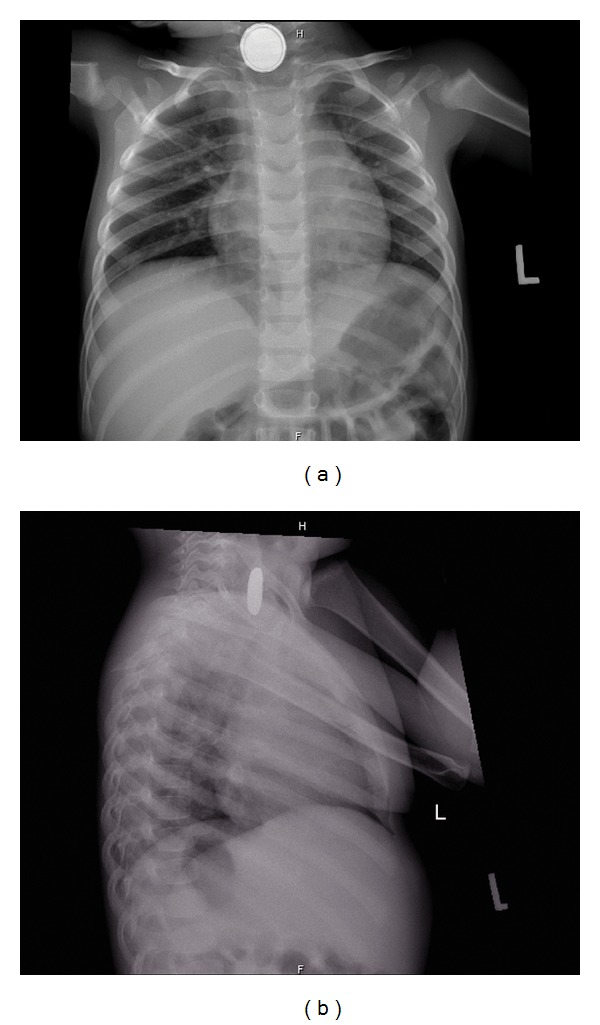
Plain X-ray: an esophageal button battery foreign body. (a) Anteroposterior view (double contour), (b) Lateral view.

**Figure 3:**
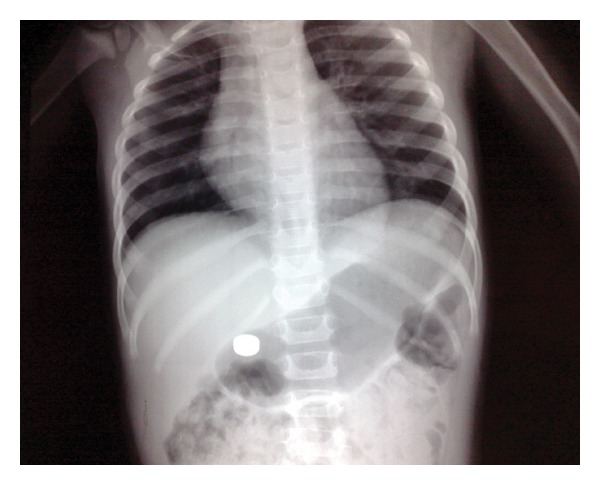
Plain X-ray, anteroposterior view: a button battery foreign body in the stomach.

Plain X-ray successfully detected radio opaque foreign bodies in all cases (Figures 1(a), 1(b), 2(a), 2(b), and 3). Button batteries appeared as a double ring or halo (double density) (Figures 1(a) and 2(a)) and had a stepped-off appearance (Figures 1(b) and 2(b)).

**Figure 4:**
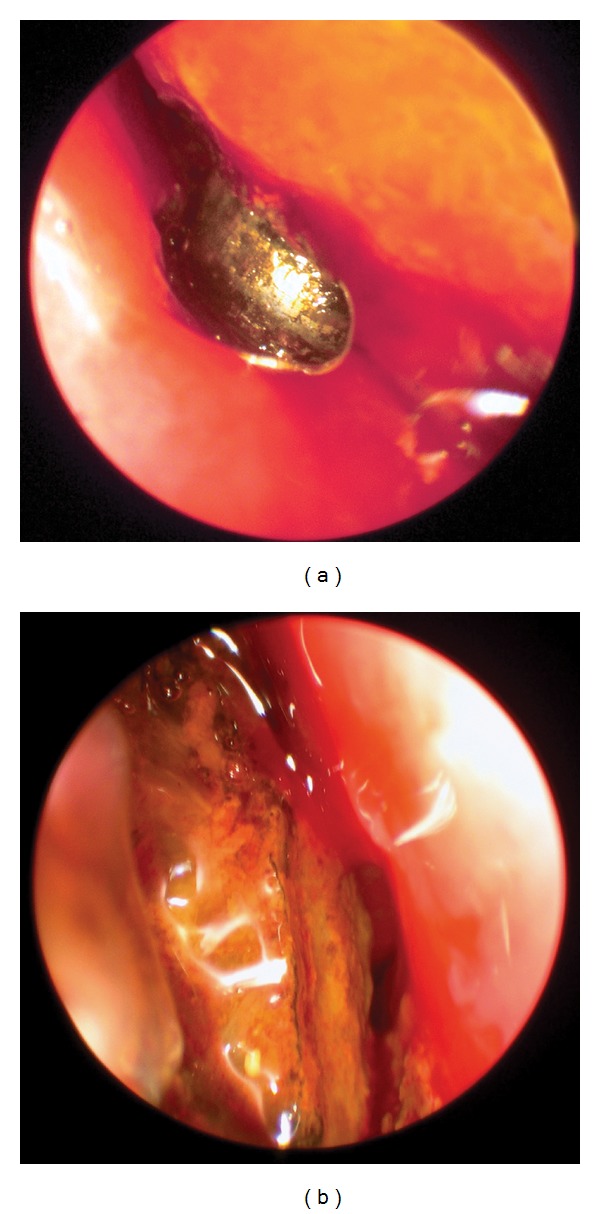
Intraoperative endoscopic view of a right nasal button battery foreign body.

**Figure 5:**
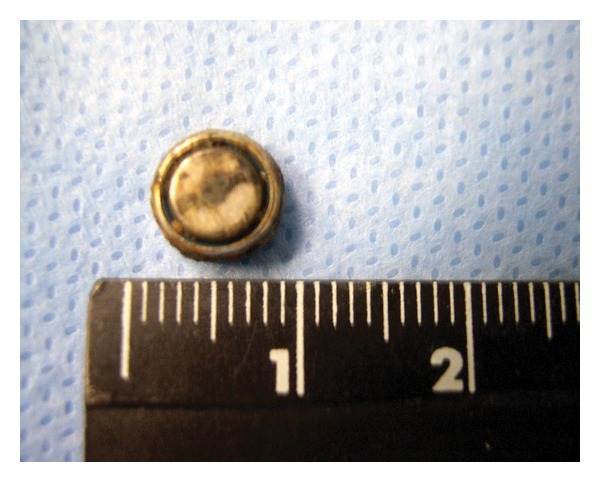
An extracted button battery from the nose (8 mm).

Nasal endoscopy was used for examination of the nasal cavity and removal of the button batteries (Figures 4(a), 4(b), and 5). One patient had an anterior medium sized nasal septal perforation, three patients had mucosal turbinate and septal ulcerations, and one patient had necrosis of the inferior turbinate (Table 1). All crusts and necrotic tissues were debrided. The nasal cavity was cleaned, and silastic splints with antibiotic ointment were placed for two weeks to prevent nasal adhesions. Postoperative treatment included oral antibiotics and physiological saline nasal spray. 

**Figure 6:**
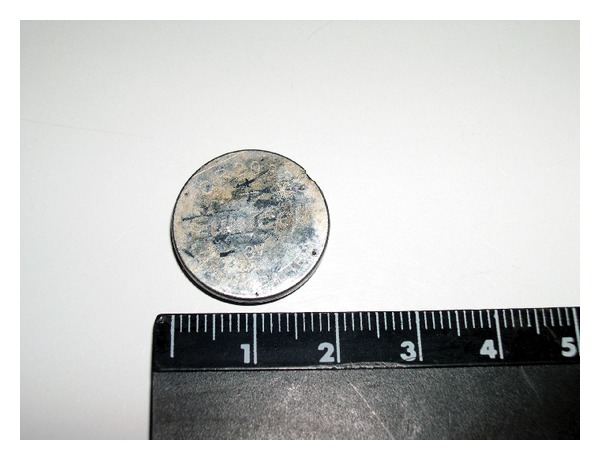
An extracted button battery from the esophagus (20 mm).

Rigid esophagoscopy was used for removal of the esophageal button batteries (Figure 6). They were impacted in the cervical esophagus, and they stayed in place for 2–6 hours. One patient had mucosal edema and discoloration; one patient had a mucosal burn while two patients had mucosal burns with necrotic tissues that were gently debrided (Table 1). Rigid bronchoscopy and esophagoscopy revealed no other abnormalities. No esophageal perforation was detected. A nasogastric tube was inserted under vision. Postoperative treatment included nasogastric tube feeding for 10 to 14 days, steroids, intravenous antibiotics, and proton pump inhibitors. A barium swallow was done before allowing the child to eat. It was repeated after three months, and it revealed no stricture formation. 

For patients with a button battery in the stomach (Figure 3), they had good general condition with stable vital signs. They had no abdominal pain or signs of peritonitis. The patients were observed and expectantly managed. Oral antacids and prokinetics are given. They were followed up by serial plain X-ray abdomen. The button battery was detected in their stool within two days.

For an impacted button battery in the ear, the patient presented three days after inserting it into his left ear. His parent gave a history of a failed trial of removal in an outpatient clinic. He presented with otalgia and serosanguinous otorrhea. Otoscopy revealed a button battery in the bony part of the external auditory meatus with proximal meatal edema and erythema. Under general anesthesia, with the use of the operative microscope, the button battery was removed revealing a medium sized central tympanic membrane perforation. The necrotic skin tissue and crusts were debrided, and a pack with antibiotic and steroid ointment was inserted in the external ear canal for one week. Pure tone audiometry was done, and it revealed mild conductive hearing loss. Three months postoperatively, the left external canal was normal and a small central tympanic membrane perforation remained. 

The average follow-up period was 4.3 months with a range from 3 to 7 months. None of the patients was lost to follow up.

## 4. Discussion

The recent development of technology has accelerated broad use of button batteries. They are used to power various electronic devices and are increasingly used in day to day life [5, 6]. The first reported case of a button battery foreign body was in 1977 and involved a child who swallowed a camera battery which lodged in the proximal esophagus [7]. Batteries account for less than 2% of the foreign bodies ingested by children [8–10]. Over the last two decades, the ingestion of button batteries is, unfortunately, becoming an increasingly common problem faced in the pediatric practice. It is mainly seen in the young children, with a peak incidence between six months and three years [11–14]. The annual incidence of battery ingestions reported to United States poison centers from 1985 to 2009 fluctuated up and down between 6.3 and 15.1 cases per million population. Thirteen deaths related to tissue damage in the esophagus or airway, and 73 major outcomes were described [15]. 

With the production of increasingly small sized batteries for use in miniaturized electronic equipments and toys, the incidence of impaction of batteries in previously uninvolved orifices such as the nose and ear appears to be on the rise [16, 17]. 

Based on their chemical composition, five types of batteries are in common use: manganese, silver, mercury, lithium, and zinc [10]. The vast majority of button batteries today are of the alkaline variety [1]. 

In the literature, four mechanisms of injury have been suggested: (1) leakage of the battery contents with direct corrosive damage, (2) direct electrical current effects on the mucosa and resultant mucosal burns, (3) pressure necrosis resulting from prolonged local pressure on the tissue, and (4) local toxic effect due to absorption of substances: this can be the case in mercuric oxide batteries [3, 17–20]. Exudation of tissue fluids caused by a burn injury creates a moist environment. In vitro studies have shown that spontaneous leakage of electrolyte solution occurs when alkaline batteries are exposed to moisture. The leaked alkaline electrolyte solution can penetrate deeply into tissues producing a liquefying necrosis. This results in dissolution of protein and collagen, saponification of lipids, dehydration of tissue cells, and consequential extensive tissue damage [6]. 

Only few hours may be needed to result in major complications; therefore, button battery impaction must be distinguished from impaction of other foreign bodies and consequently approached differently [11]. The key to proper management of button battery foreign bodies is rapid diagnosis and removal of any object lodged in the ear, nose, and upper aerodigestive tract that is suspicious for a button battery. Occasionally, the ingestion or placement of the battery is witnessed, and the child is promptly brought to the hospital for treatment. However, the exact nature of the foreign body is often unknown or mistaken. Symptoms are variable. Some children may present with no signs or symptoms while others can have nonspecific signs like pain, cough, vomiting, irritability, fever, and tachycardia. More specific symptoms include drooling, poor oral intake, epistaxis, rhinorrhea, and foul otorrhea. Despite assurances from the parents that button batteries were not available and lack of symptoms, it is essential to rule out the possibility of any foreign body being a button battery [1]. 

Delayed diagnosis of an impacted battery is not uncommon and may occasionally present as a long term serious complication [21]. Therefore, a change in the clinical approach to button battery foreign bodies is required to avoid misdiagnosis or delayed treatment. 

In this study, the standard radiologic workup for a suspected battery foreign body was immediate nose, neck, chest, and abdominal plain X-ray films, in anteroposterior and lateral views. Plain X-ray films have high availability, low costs, and high accuracy in outlining radio opaque objects [21]. Button batteries should always be diagnosed if a proper X-ray with adequate exposure is taken. They have a distinctive appearance on radiography as they have a bilaminar structure, making them appear as a double ring or halo (double density) on anteroposterior view and a step-off at the separation between the anode and cathode on lateral view. Small batteries have a more subtle contour which is hard to detect. When in doubt, repeated X-ray films in different angles are advised to achieve a correct diagnosis [21–23]. 

Occasionally, coins may mimic the shape, size, and contour of batteries, which make them undistinguishable. If a battery is diagnosed as a coin on plain film, it may delay its removal unnecessarily [21]. Coin cell batteries typically differ from coin currency on radiographs by appearing slightly more translucent, having an enhanced rim, and showing a step-off on lateral view while a coin has a sharp and crisp edge [2, 23]. Correlation of the suspected objects as described by the patient himself or his caregivers to the radiologic findings is important, in order to plan carefully the next steps in management [21]. 

In the literature, there are reports of significant morbidity caused by button battery ingestion and button batteries in the ear or nose [1]. The consequences are determined largely by the site at which the battery is lodged, the duration it remains in situ, the battery size, its age (new or old), its power, and the possibility of heavy-metal absorption [21, 24–26].

In this study, five patients had a nasal button battery. One patient developed a nasal septal perforation while one patient developed necrosis of the inferior turbinate. Nasal button battery impaction may produce mucosal turbinate and septal ulceration in as little as three to six hours. Necrosis of the inferior turbinates has occurred at 24 hours [18]. Inferior meatus ulceration, saddle deformities, chondritis, atrophic rhinitis, alar collapse, septal perforation, and nasal/choanal stenosis may ultimately result [1, 15]. Therefore, button batteries must be removed from the nose immediately because of the danger of liquefaction necrosis of the surrounding tissue. Dislodgement, debridement, and cleaning are best accomplished in the operating room under general anesthesia with immediate placement of stents for severe necrosis to prevent the development of nasal adhesions. 

This work included one patient with an impacted button battery in the external ear canal. He presented late and developed tympanic membrane perforation. Ear irrigation should be avoided because the electric current and/or battery contents can cause a liquefaction tissue necrosis [16]. Delay in the removal of a button battery could potentially lead to stenosis of the external auditory canal, ossicular erosion, facial nerve injury, and necrosis of the medial wall of the middle ear resulting in a sensorineural deafness and damage to the vestibular labyrinth [1, 6].

In this study, three patients presented with a button battery in the stomach that passed uneventfully while four patients had an impacted esophageal button battery. Our primary therapeutic strategy was endoscopic removal and examination in order to assess the damage in the esophagus and tailor treatment accordingly. None of the patients developed an esophageal perforation. 

Most cases of button battery ingestion end uneventfully. However, those batteries that lodge in the esophagus can result in serious complications and even death [22]. Patients with batteries lodged in the esophagus have a greater potential for a serious outcome than those who have batteries that pass into the distal gastrointestinal tract. This is because batteries impacted in the esophagus exert a cumulative effect in a localized area without the benefit of dilution of chemical and electrical effects provided by the gastrointestinal secretions in more distal segments [11].

 Impaction in the esophagus has been noted to most frequently occur in patients younger than 5 years old, with smaller esophageal diameter, and often occur with battery diameter larger than 20 mm. The larger the diameter of the battery, the more likely it is to lodge in the esophagus [21, 23]. Severe esophageal damage may occur in a very short period of time. Esophageal corrosive injury and burn can occur as early as 2.5 hours after ingestion, while esophageal perforation can occur after as short a time as of five hours [22, 23]. Other complications include tracheoesophageal fistula, mediastinitis, and perforation of the aorta. Furthermore, airway compromise from esophageal edema has been reported. Esophageal stenosis may be detected few months after removal of the foreign body [23, 27, 28]. 

Thus, for patients with batteries lodged in the esophagus, removal is urgently needed within two hours while batteries that are in the stomach or beyond in an asymptomatic patient should be left to pass spontaneously with inspection of the stool or possible repeat radiography in 10 to 14 days to confirm passage [29]. 

Prevention of button battery foreign bodies, especially ingestion, is essential. Prevention focuses on checking and securing the battery compartment of all household products, storing batteries out of a child's reach and sight, never leaving batteries sitting out loose, and not allowing children to play with batteries. Furthermore, product manufacturers need to redesign battery-powered household products to secure the battery compartment [15]. 

## 5. Conclusion and Recommendations

Button batteries are now ubiquitous to our culture. The button battery is a hazardous material and should be treated as a life-threatening foreign body due to its electrochemical composition and the large potential for local damage and severe mucosal injuries. The potential for rapid tissue destruction mandates prompt removal of the button batteries. Therefore, to improve outcomes, early detection is the key in the management of button battery foreign bodies. An urgent initial radiography is required. Radiologists must be aware of its danger and be trained to differentiate the button batteries from the coins. Radiographs should be examined for the battery's double-rim or halo effect on the anteroposterior view or step-off on the lateral view. Physicians must recognize the hazardous potential and serious implications of such an accident and must consider the diagnosis (particularly in unwitnessed ingestion). Button batteries that are lodged in the esophagus pose the greatest risk, requiring prompt removal. Endoscopic removal of esophageal batteries is essential to determine the extent of injury and anticipate complications. 

The most effective management strategy is prevention. The parents and child care providers should be educated about the potential hazards associated with battery exposure so they will be aware of its dangerous nature. The products containing button batteries are either kept away from children or the batteries are secured safely in the product. The public education for this serious problem is necessary as increased public awareness through the public health and health care providers could reduce exposure to and injuries from these batteries. Industry changes, including improved packaging and button battery markings, will also contribute to reduce morbidity in children. The primary prevention of battery foreign bodies would be more effective than improved treatment.

## Figures and Tables

**Table 1:** The results summary.

Sex	Age (month)	Site of impaction of BB	Duration of impaction of BB	Size of BB (millimeter)	Type of BB	Complication	Follow-up period (month)
Male	28	Left nasal fossa	1 day	15	Alkaline	Mucosal turbinate and septal ulcerations	3
Male	50	Left nasal fossa	18 hours	8	Alkaline	Mucosal turbinate and septal ulcerations	3
Female	48	Right nasal fossa	12 hours	12	Alkaline	Mucosal turbinate and septal ulcerations	3
Male	34	Right nasal fossa	3 days	10	Alkaline	Necrosis of the inferior turbinate	4
Male	53	Left nasal fossa	1 week	12	Alkaline	Nasal septal perforation	6
Female	40	Upper esophagus	2 hours	23	Alkaline	Mucosal edema and discoloration	5
Female	42	Upper esophagus	5 hours	22	Alkaline	Mucosal burns and necrotic tissues	7
Male	30	Upper esophagus	4 hours	22	Alkaline	Mucosal burns	6
Male	23	Upper esophagus	6 hours	20	Alkaline	Mucosal burns and necrotic tissues	7
Male	36	Stomach	3 hours	15	Alkaline	—	3
Male	23	Stomach	2 hours	12	Alkaline	—	3
Female	24	Stomach	5 hours	15	Alkaline	—	3
Male	38	Left external ear	3 days	8	Alkaline	Tympanic membrane perforation	3

The button battery: BB.
